# The influence of nutritional state on the fatty acid composition of circulating lipid fractions: implications for their use as biomarkers of dietary fat intake

**DOI:** 10.48101/ujms.v126.7649

**Published:** 2021-07-13

**Authors:** Sion A. Parry, Fredrik Rosqvist, Sarah Peters, Rebecca K. Young, Thomas Cornfield, Pamela Dyson, Leanne Hodson

**Affiliations:** a Oxford Centre for Diabetes, Endocrinology and Metabolism, University of Oxford, Churchill Hospital, Oxford, United Kingdom; b Department of Public Health and Caring Sciences, Clinical Nutrition and Metabolism, Uppsala University, Uppsala, Sweden; c National Institute for Health Research Oxford, Biomedical Research Centre, Oxford University, Hospital Trusts, Oxford, United Kingdom

**Keywords:** Postprandial, fatty acids, biomarker, lipid fractions, fatty acid composition

## Abstract

**Background:**

The fatty acid (FA) composition of blood can be used as an objective biomarker of dietary FA intake. It remains unclear how the nutritional state influences the FA composition of plasma lipid fractions, and thus their usefulness as biomarkers in a non-fasted state.

**Objectives:**

To investigate the associations between palmitate, oleate and linoleate in plasma lipid fractions and self-reported dietary FA intake, and assess the influence of meal consumption on the relative abundance of these FA in plasma lipid fractions (i.e. triglyceride [TG], phospholipids [PLs] and cholesterol esters [CEs]).

**Design:**

Analysis was performed in plasma samples collected from 49 (34 males and 15 females) participants aged 26–57 years with a body mass index (BMI) between 21.6 and 34.2 kg/m^2^, all of whom had participated in multiple study visits, thus a pooled cohort of 98 data sets was available for analysis. A subset (*n* = 25) had undergone nutritional interventions and was therefore used to investigate the relationship between the FA composition of plasma lipid fractions and dietary fat intake.

**Results:**

Significant (*P* < 0.05) positive associations were observed between dietary polyunsaturated fat and linoleate abundance in plasma CE. When investigating the influence of meal consumption on postprandial FA composition, we found plasma TG palmitate significantly (*P* < 0.05) decreased across the postprandial period, whereas oleate and linoleate increased. A similar pattern was observed in plasma PL, whereas linoleate abundance decreased in the plasma CE.

**Conclusion:**

Our data demonstrate that the FA composition of plasma CE may be the lipid fraction to utilise as an objective biomarker when investigating recent (i.e. previous weeks-months) dietary FA intakes. In addition, we show that the consumption of a high-fat meal influences the FA composition of plasma TG, PL and CE over the course of the postprandial period, and therefore, suggest that fasting blood samples should be utilised when using FA composition as a biomarker of dietary FA intake.

## Introduction

The high prevalence of metabolic diseases such as cardiovascular disease (CVD) and type 2 diabetes (T2D) are recognised as a global health issue (1). The relationship between dietary fat quantity and quality and metabolic health is highly debated with some suggesting that increased intake of saturated fat is associated with an increased risk for the development of cardiometabolic diseases (e.g. CVD and T2D), whereas others suggest that no relationship exists (2–5). The conflicting findings may be partly attributable to the methods of dietary assessment used in epidemiological studies, which typically involve the use of food-frequency questionnaires or food diaries, which have known limitations (6–8). Using the fatty acid (FA) composition of blood and tissues as a biomarker of dietary FA intake is an additional and objective method of dietary assessment (9, 10). Typically, the FA composition of lipid fractions in blood is measured in the fasting state to avoid the potentially confounding influence of recent dietary FA intake (11, 12). However, although it is often assumed that non-fasting samples cannot be used to investigate biomarkers of dietary FA intake, this assumption has not been fully investigated; it remains unclear as to whether nutritional state influences the FA composition of circulating lipid fractions. For large-scale observational studies, where it can be logistically challenging to obtain fasting samples, it would be useful to determine whether nutritional state influences circulating FA composition as it may potentially reduce the burden on researchers and participants. Although a number of observational studies have previously obtained non-fasted samples from participants (13–17), or have obtained samples after only a relatively short fasting period (minimum of 4 h fasting) (18, 19), it remains unclear whether the presence of recently ingested fat influenced their findings. Therefore, the aim of this study was to investigate: (1) the association between the dietary saturated (SFA), monounsaturated (MUFA) and polyunsaturated (PUFA) FAs in plasma lipid fractions and self-reported dietary FA intakes, and (2) the influence of meal consumption on the relative abundance of specific SFA, MUFA and PUFA in plasma lipid fractions across a 6-h postprandial period.

## Materials and methods

### Participants

Participants were recruited from the Oxford Biobank (www.oxfordbiobank.org.uk) (20) or from the wider Oxfordshire area through advertisement. Based on data provided at screening, all volunteers were non-diabetic and free from any known disease, were not taking medication known to affect lipid or glucose metabolism, and did not consume alcohol above recommended limits. Some, but not all, of the data reported in this work constitute a reanalysis of previously published studies (21, 22) and ongoing dietary intervention trials (ClinicalTrials.gov identifiers: NCT03090347 and NCT03587753). Data in this manuscript were from a total of 49 (34 males and 15 females) participants aged 26–57 years with a body mass index (BMI) between 21.6 and 34.2 kg/m^2^ (Table 1). Twenty-five participants were enrolled in one of two dietary intervention studies, both of which involved changing their relative intakes of fat and carbohydrate. The 25 participants were representative of the study population, as they were aged 38–54 years, with a BMI between 22.0 and 34.2 kg/m^2^. Data from this subset were used to investigate the relationship between dietary FA intake and the fasting FA composition of circulating plasma lipid fractions. However, within this subset, four food diaries were missing or incomplete, leaving 46 complete sets of data for analysis.

**Table 1:** Baseline characteristics of participants.

Age (years)	46±7
Sex (M/F)	34/15
BMI (kg/m^2^)	26.6±3.1
Glucose (mmol/L)	5.0±0.6
Insulin (mU/L)	8.3±4.5
HOMA-IR	1.9±1.1
Total cholesterol (mmol/L)	4.3±0.7
HDL cholesterol (mmol/L)	1.2±0.4
Triglycerides (TGs) (mmol/L)	0.9±0.4
Non-esterified fatty acid (µmol/L)	519±275

Data are mean ± SD. *n* = 49.

M, male; F, female; HDL, high-density lipoprotein; HOMA-IR, Homeostatic Model Assessment for Insulin Resistance.

Of the total 49 participants, the remaining 24 participants were enrolled in a randomised crossover study, involving two postprandial study days separated by a 2-week washout period, during which they were asked to maintain their habitual diet and physical activity patterns (21). As all participants (*n* = 49) included in this study took part in two postprandial study visits, this gave a total of 98 data sets to investigate the temporal changes in plasma FA composition in response to meal consumption. A flow chart of participant recruitment and experimental procedures is presented in Supplementary Fig. 1.

All studies were approved by the respective Research Ethics Committee, and all subjects provided written informed consent. Prior to study days, subjects were asked to refrain from strenuous physical activity and to not consume alcohol for a minimum of 24 h. After an overnight fast, subjects came to the Clinical Research Unit at the Oxford Centre for Diabetes, Endocrinology and Metabolism, and a fasting venous blood sample was taken. Subjects were then given a standardised test meal, and venous blood samples were taken at regular intervals for 6 h after meal consumption.

### Dietary interventions and assessments

A subset (*n* = 25) of participants included in this study underwent dietary interventions. Of these, 16 participated in a randomised crossover trial in which they underwent two dietary interventions: 1) a 4-week low-fat, high-carbohydrate diet enriched in free-sugars and 2) a 4-week high-fat, low-carbohydrate diet enriched in SFA (22). The remaining nine participants were included in an ongoing study (NCT03090347) and underwent either a 2-week low-fat, high-carbohydrate diet enriched in free-sugars (*n* = 8) or a 2-week high-fat, low-carbohydrate diet enriched in SFA (*n* = 1) (Supplementary Fig. 1).

Dietary intakes were assessed by food diaries collected on 3 days, including a weekend day. Participants taking part in dietary interventions were instructed to maintain their usual body weight, physical activity levels and alcohol intakes throughout the intervention periods, and were contacted weekly by a member of the research team to aid adherence. Dietary intakes were analysed using the Nutritics dietary analysis online software (Dublin, UK), by a registered dietitian to determine energy and nutrient intakes.

### Standardised test meal

On the study day, participants consumed a standardised test meal consisting of 40 g cereal (Kellogg’s Rice Krispies), 200 g skimmed milk and a chocolate drink containing 40 g oil, providing 591 kcal, with ~64% energy as fat, ~30% energy as carbohydrate and ~6% energy as protein. The oil used was either olive oil (Meal A) or 15 g sunflower oil plus 25 g palm oil (Meal B). The FA composition of Meal A was ~13% palmitate, ~64% oleate, ~11% linoleate, ~3% stearate and ~9% minor FA, whilst the FA composition of Meal B was ~32% palmitate, ~35% oleate, ~27% linoeate, ~5% stearate and ~1% minor FA. All subjects consumed the same meal twice, separated by a period of 2–11 weeks.

### Analytical procedures

Whole-blood was collected into heparinised tubes, and plasma was immediately separated for analysis by centrifugation at 4°C. Plasma glucose, triglycerides (TGs), total cholesterol and high-density lipoprotein (HDL) cholesterol were analysed enzymatically (Ilab 600/650 Clinical Chemistry, Werfen).

### Fatty acid composition

Total lipids were extracted from plasma by using chloroform–methanol (2:1, v/v) (23), and plasma lipid fractions (TG, phospholipid [PL] and cholesterol esters [CEs]) were separated by solid-phase extraction (24), followed by methylation using acidified methanol. The FA profile of extracted samples was then determined via gas chromatography with flame ionisation detection (25). FAs were identified by comparing sample retention times to a known standard, and results are expressed as mol%.

### Statistical analysis

Data were analysed using SPSS (version 25.0). The specific FAs investigated were restricted to the major dietary SFA, MUFA and PUFA (i.e. palmitate, oleate and linoleate, respectively). Normality of variables was assessed by Shapiro–Wilk test and visually by histograms. The Spearmans rank correlation coefficient was used to assess the associations between the specific FAs in lipid fractions and the relative percentages of energy intake from SFA, MUFA and PUFA calculated from 3-day diet diaries. Differences in FA abundance in response to meal consumption were analysed using one-way repeated measures (time) ANOVA. Where a significant main effect of time was noted, Bonferroni post-hoc comparisons were made for postprandial vs. fasting time points (0 min). Data are presented as mean and standard deviation (SD).

## Results

### Relationship between dietary FA intake and the FA composition of circulating plasma lipid fractions

**Figure 1:**
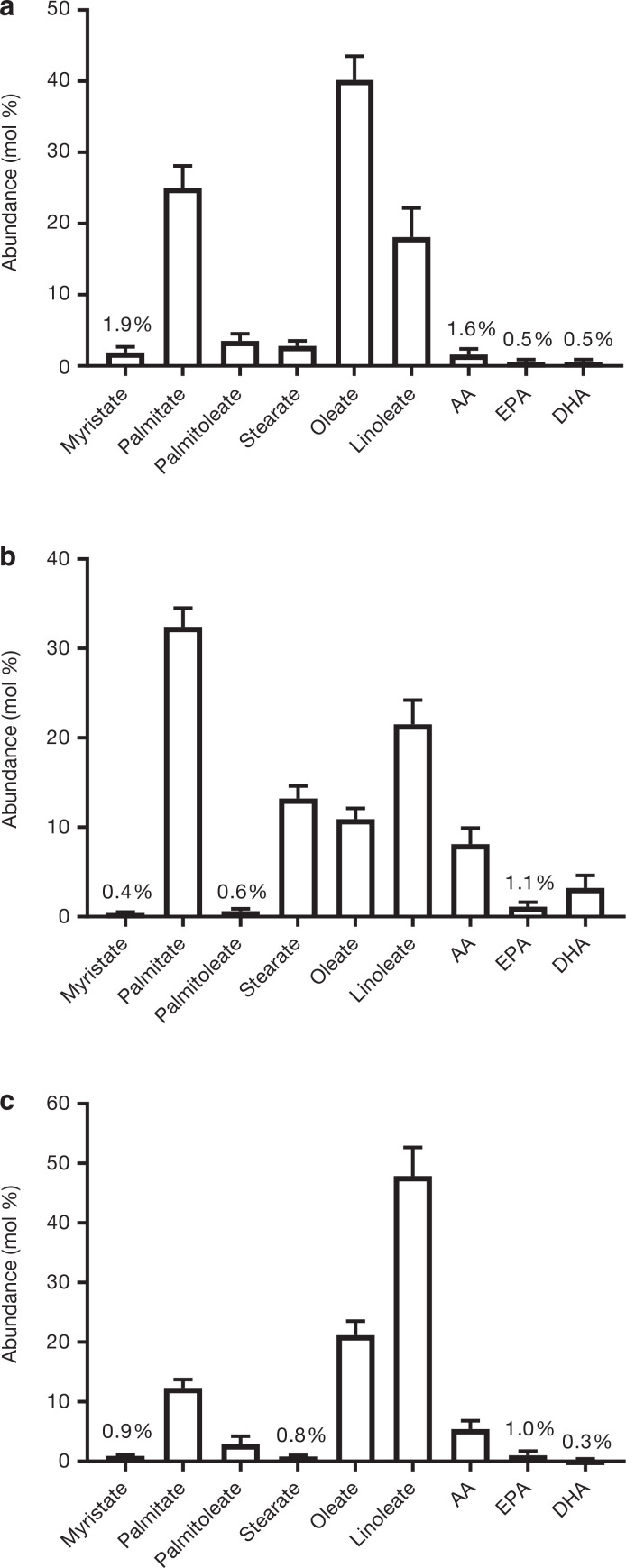
Fasting plasma FA composition for (a) TG (*n* = 98), (b) PL (*n* = 76) and (c) CE (*n* = 96). AA, arachidonic acid; EPA, eicosapentaenoic acid; DHA, docosahexaenoic acid. Data are mean ± SD.

The fasting FA composition of plasma TG, PL and CE fractions is presented in Figure 1. As data were obtained from participants undertaking various dietary interventions, some of which involved increasing dietary carbohydrates/free sugars, and some increasing dietary fat/saturated fat, there were wide variations in self-reported intakes of total fat, SFA, MUFA and PUFA, which is reflective of the specific dietary interventions which were undertaken (Table 2). We assessed the association between dietary FA intake and the abundance of specific FA that represent the major dietary SFA, MUFA and PUFA sources, in the respective plasma lipid fractions. We found no significant associations between dietary FA and the abundance of palmitate, oleate and linoleate in plasma TG or PL (Table 3). However, significant (*P* < 0.05) positive associations were observed between dietary PUFA and linoleate in plasma CE, whilst there were no associations between dietary SFA and the abundance of plasma CE palmitate, or dietary MUFA and plasma CE oleate (Table 3).

**Table 2:** Dietary fat intake as a proportion of total energy (TE) intake.

Total fat (%TE)	31.5±13.7
Saturated fat (%TE)	12.3±7.0
Polyunsaturated fat (%TE)	4.7±2.5
Monounsaturated fat (%TE)	10.5±5

Data are mean ± SD. *n* = 46.

**Table 3:** Spearmans rank correlation coefficients between the abundance of palmitate, oleate, and linoleate in circulating lipid fractions and the relative percentages of energy intake from dietary saturated fatty acids (SFA), polyunsaturated fatty acids (PUFA) and monounsaturated fatty acids (MUFA).

Mol (%)	Dietary SFA (%TE)	Dietary MUFA (%TE)	Dietary PUFA (%TE)
Triglyceride (TG) palmitate	-0.029		
Phospholipid (PL) palmitate	-0.003		
Cholesterol ester (CE) palmitate	0.031		
TG oleate		-0.057	
PL oleate		-0.364	
CE oleate		-0.210	
TG linoleate			0.211
PL linoleate			0.198
CE linoleate			0.372*

* *P* < 0.05. *n* = 46 for TG, *n* = 27 for PL and *n* = 44 for CE.

### Temporal changes in FA composition following meal consumption

As FA composition is typically measured in the fasting state, we investigated whether the consumption of a meal influences the relative abundance of palmitate, oleate and linoleate in circulating lipid fractions. We achieved this by feeding participants a high-fat test meal, of known FA composition, and assessing changes in plasma palmitate, oleate and linoleate over the course of the postprandial period in the respective lipid fractions.

**Figure 2:**
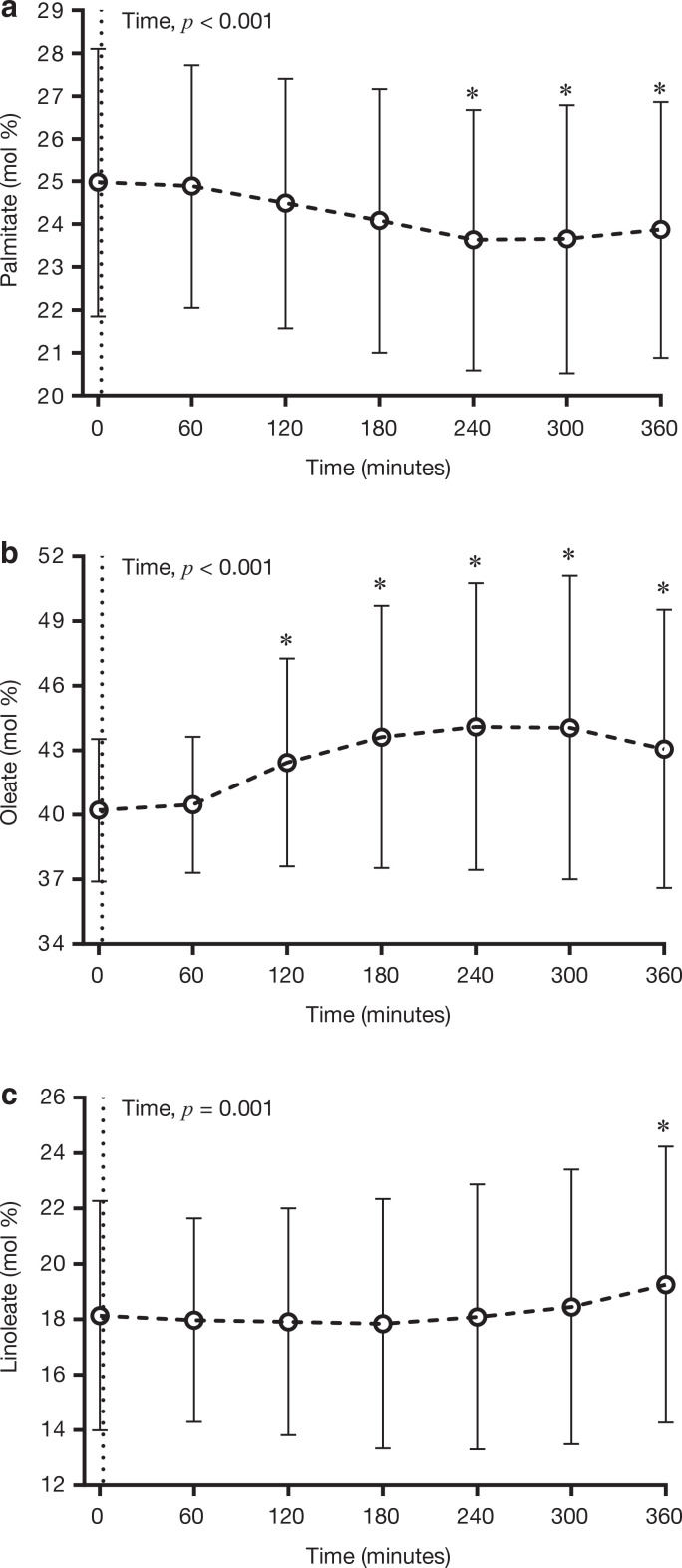
Temporal changes in the relative abundance of (a) palmitate, (b) oleate and (c) linoleate in plasma TG in response to the consumption of a high-fat test meal. Data are presented as mean ± SD. *n* = 98. *P < 0.05 compared to fasting (Time 0). The dotted line at Time 0 denotes the consumption of the experimental test meal.

The consumption of the test meal significantly (*P* < 0.05) decreased the abundance of palmitate in plasma TG, with time points 240–360 min being significantly (*P* < 0.05) lower than time 0 (fasting), and the greatest differences (i.e. ~1.3 mol%) being apparent between 240 min and 0 min (Figure 2a). Conversely, meal consumption significantly (*P* < 0.05) increased the abundance of oleate, and linoleate in plasma TG, with the oleate peaking at 240 min, which was ~3.8 mol% greater than 0 min (Figure 2b), and linoleate peaking at time point 360 min, which was ~1.1 mol% greater than 0 min (Figure 2c).

**Figure 3:**
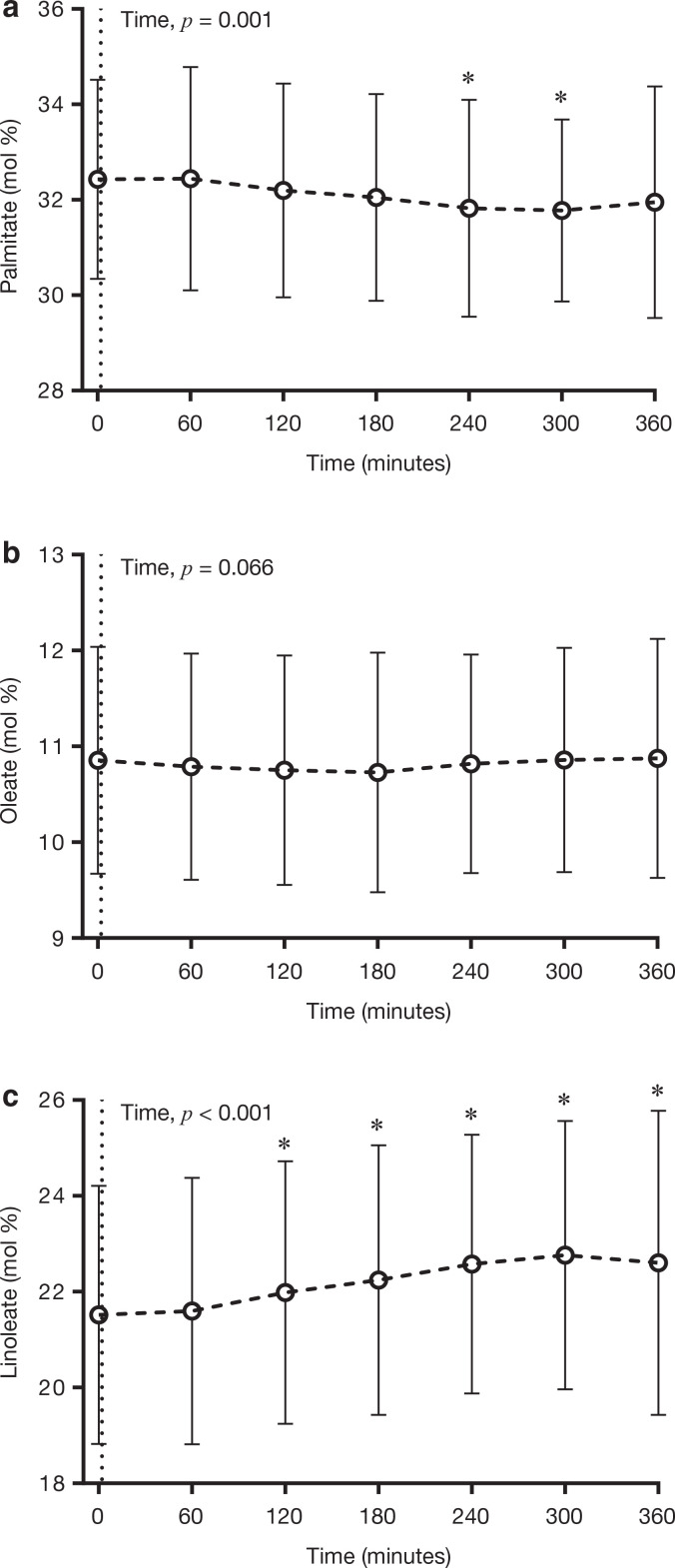
Temporal changes in the relative abundance of (a) palmitate, (b) oleate and (c) linoleate in plasma PL in response to the consumption of a high-fat test meal. Data are presented as mean ± SD. *n* = 76. *P < 0.05 when compared to fasting (Time 0). The dotted line at Time 0 denotes the consumption of the experimental test meal.

For plasma PL, the general trend was similar to the changes observed in plasma TG, although not as striking. The relative abundance of palmitate was significantly (*P* < 0.05) decreased by the consumption of the test meal, with significant differences apparent between time points 240–300 min and 0 min, with a nadir at 300 min, which was ~0.7 mol% lower than 0 min (Figure 3a). The relative abundance of oleate was not influenced by meal consumption (Figure 3b). The abundance of linoleate was significantly increased (*P* < 0.05) following meal consumption, with time points 120 min onwards significantly greater than 0 min, and peaking at 300 min (i.e. ~1.2 mol% greater than 0 min) (Figure 3c).

**Figure 4:**
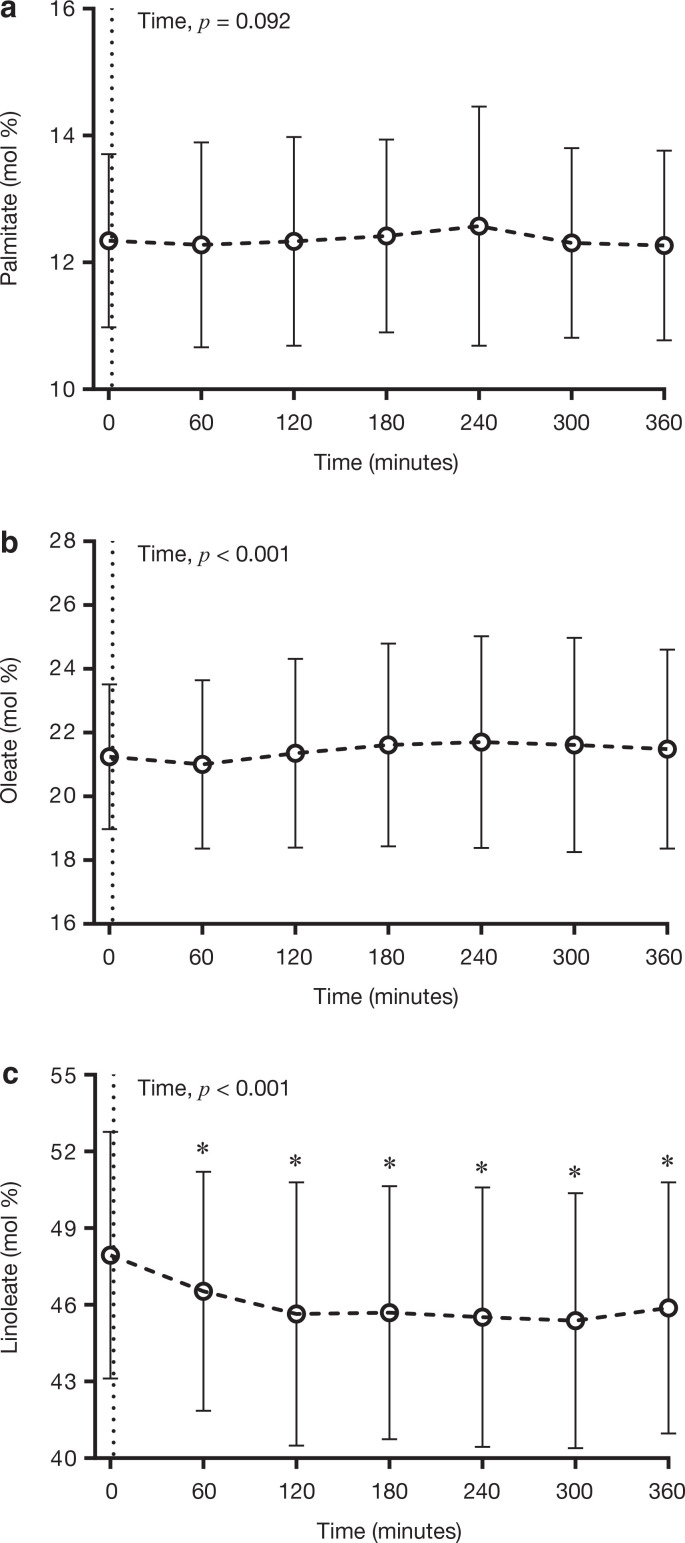
Temporal changes in the relative abundance of (a) palmitate, (b) oleate and (c) linoleate in plasma CE in response to the consumption of a high-fat test meal. Data are presented as mean ± SD. *n* = 96. *P < 0.05 when compared to fasting (Time 0). The dotted line at Time 0 denotes the consumption of the experimental test meal.

In contrast to the FA composition of plasma TG and PL, the consumption of the test meal did not influence the relative abundance of palmitate in plasma CE (Figure 4a). There was, however, a significant (*P* < 0.05) main effect of time for oleate in plasma CE, although Bonferroni post hoc comparisons revealed no statistically significant differences between any postprandial time points and 0 min (Figure 4b). The abundance of linoleate in plasma CE significantly (*P* < 0.05) decreased following meal consumption, reaching a nadir at 300 min, which was ~2.6 mol% lower than 0 min (Figure 4).

## Discussion

The FA composition of blood and tissues has frequently been used as a biomarker of dietary FA intake (9). Typically, the FA composition of blood lipids is measured in blood samples taken from individuals after an overnight fast. However, some large-scale observational studies have utilised non-fasting samples (13–17), and it remains unclear if feeding influences the postprandial FA composition of plasma lipid fractions. We therefore aimed to assess the associations between FA composition in plasma lipid fractions and dietary FA intake in participants who had undergone dietary interventions to investigate biomarkers of dietary FA intake. In addition, we aimed to determine the influence of meal consumption on the postprandial FA composition of plasma TG, PL and CE. Overall, our findings suggest that the FA composition of plasma TG or PL does not reflect dietary fat intakes over the short term (2–4 weeks), whereas the relative abundance of plasma CE linoleate was positively associated with self-reported intakes of PUFA. Thus, plasma CE linoleate may represent a valid biomarker of dietary PUFA even in situations where individuals have recently altered their dietary FA intakes. Moreover, our data indicate that the consumption of a high-fat test meal acutely influences the FA composition of plasma TG, PL and CE. Therefore, it would seem prudent to suggest that fasting blood samples should be utilised when using FA composition as a biomarker of dietary FA intake, as the consumption of a high-fat meal may confound measurements of FA composition taken in a non-fasting state.

Measurement of FA composition in various tissue and plasma lipid pools provides an objective assessment of dietary FA intake, which may strengthen data obtained from self-reported dietary records which have limitations (e.g. underreporting) (7, 8, 26). It has been suggested that due to the slow turnover of adipose tissue (i.e. half-life of ~600 days), adipose tissue FA composition may be most reflective of long-term (i.e. 2–3 years) fat intake (27–29). In contrast, the FA composition of red blood cells, plasma CE and PL has been shown to reflect dietary fat intakes within weeks (29–31). Epidemiological investigations have generally used the FA composition of various plasma/serum lipid fractions as biomarkers of dietary FA intake (32, 33), which may be because blood sampling is relatively simpler than adipose tissue biopsies. However, there is heterogeneity between the metabolism and turnover of circulating lipid fractions, which is reflected in their FA composition, and debate continues as to which plasma lipid fraction represents the most accurate biomarker of dietary fat intake. We therefore investigated the relationship between self-reported dietary fat intake and the relative abundance of palmitate, oleate and linoleate (representative of the major SFA, MUFA and PUFA) in plasma TG, PL and CE.

In line with previous observations (9), we found a positive association between the abundance of linoleate in plasma CE and dietary PUFA intake, but observed no significant associations relative abundance of palmitate or oleate in plasma CE and dietary SFA and MUFA, respectively. We also observed no associations between specific FAs in the plasma TG or PL fraction and self-reported intake of dietary SFA, MUFA and PUFA. It is plausible that positive associations are observed more often for PUFA than other FAs as linoleate represents an essential FA, whereas the *in vivo* synthesis of palmitate and oleate may influence their circulating abundance independently of dietary intake. Equally, palmitate and oleate are relatively ubiquitous in foods, which, when combined with the inability of FA composition measures to establish quantitative intakes, may make it challenging to separate individuals with low and high intakes of these FA. When comparing the utility of FAs in various lipid fractions as biomarkers of dietary FA intake, Furtado et al. (12) reported that combining plasma TG and non-esterified FA (NEFA) fractions was more reflective of dietary FA intake than total plasma, CE or PL FA composition. The difference between our findings and those of Furtado et al. (12) is likely due to differences in study design. Participants in our study completed 2–4 weeks dietary interventions at the time of assessment, whereas those studied by Furtado et al. (12) had not undertaken a dietary intervention and were consuming their habitual diet, which was assessed using a food frequency questionnaire examining intakes over the previous year. It is therefore plausible that the strength of the correlation for the combined TG and NEFA fraction was driven by NEFA FA composition, which has been suggested to be reflective of adipose tissue FA composition (34).

We found that the abundance of palmitate, oleate and linoleate in plasma TG, PL and CE was influenced by a high-fat meal in a manner which reflected, to a degree, the FA composition of the test meal. Changes in FA abundance were specific to lipid fractions. The plasma TG and PL fractions were influenced to a greater extent than plasma CE. It is unsurprising that the composition of circulating TG was altered during the postprandial period as ingested FAs are initially incorporated into chylomicron-TG prior to entering the circulation (35), and the incorporation of dietary FA into hepatic very low-density lipoprotein (VLDL) TG also occurs relatively soon after ingestion (25). Thus, our data are in-line with others who have previously demonstrated that changes in the non-fasting FA composition of plasma TG are reflective of the recently ingested meal FA composition (36–38). We also show that the FA composition of plasma PL and CE is influenced by the intake of a high-fat mixed meal with significant differences apparent from 60-min onwards, dependent on the fraction and FA. Recently, Shokry et al. demonstrated that the abundance of some FA in plasma TG and NEFA, including linoleate, changed during a 7-h postprandial period following a high-calorie mixed macronutrient test meal (45 g of fat and 97 g of carbohydrate); the plasma PL FA composition was unaffected (38). This finding is in contrast to our observations, but may be explained in part by the difference in the FA composition of the meals, with Shokry et al. feeding a test meal containing 26.4 g of linoleate (i.e. over 50% of the fat component). Meal consumption has also been shown to influence the FA composition of plasma PL when assessed using lipidomic methodologies (39, 40). Our observations are in line with Karupaiah et al. who reported changes in plasma CE within 7-h, which reflected the fat composition of the consumed meal (41). Thus, regardless of methodology (GC or lipidomics), it would seem that changes in both PL and CE species have been observed in the non-fasting compared with the fasting state, but the degree and manner of change may be dependent on the composition of the test meal.

In the present work, the abundance of linoleate increased in the TG and PL fractions but decreased in the CE. These differences may be explained by differences in FA incorporation time between the fractions, as the synthesis of CE involves the enzymatic transfer of an FA from PL and cholesterol precursors, typically from the sn-2 position of PL, which is commonly occupied by a PUFA (9). Using stable isotope tracer methodology, we have previously shown the incorporation of linoleate in plasma PL is greater than palmitate following a high-fat mixed meal (21), demonstrating the metabolic heterogeneity of specific FA in lipid fractions. It is therefore plausible that the incorporation time of linoleate in plasma CE from PL is longer than we have investigated, and that the abundance of linoleate in plasma CE may have increased at a time point later than the 6-h period examined here.

Our study has a number of limitations, including: all subjects were free from known metabolic disease, and results may therefore not be reflective of other metabolic phenotypes. For instance, individuals with metabolic-associated fatty liver disease (MAFLD) demonstrate increased *de novo* lipogenesis (DNL) relative to their non-MAFLD counterparts (42), which may lead to an increased palmitate abundance in plasma lipid fractions. Participants consumed a single test meal, which was high in fat; therefore, we cannot exclude the possibility that non-fasting FA composition would change further if we had given a subsequent/second meal (i.e. more reflective of habitual dietary pattern in most individuals). Moreover, it remains unclear if the responses observed were mediated by the quantity, along with the quality of FA in the meal; it could be speculated that a lower fat meal may result in less notable/obvious responses/changes. Our test meals were devoid of marine n-3 FA; thus, we are unable to comment on the stability of these FA across the postprandial period, but it would be of interest to investigate this given their usefulness as biomarkers (43–45). Similarly, we only assessed the most abundant SFA, MUFA and PUFA; therefore, our findings cannot be extrapolated to FA of lower abundance, for example, myristate, pentadecanoic acid, stearate and arachidonic acid. We did not assess the FA composition of plasma NEFA, which whilst being a potentially good marker of long-term dietary FA intake (as it reflects AT FA composition) (9), likely does not reflect short- to medium-term dietary intake. In addition, we and others have previously shown that during the postprandial period chylomicron-derived dietary FA spillover contributes 10–50% of FA within the systemic NEFA pool (46–48). Thus, similar to plasma TG FA composition with meal consumption, the FA composition of plasma NEFA would be highly influenced over the postprandial period by the fat content and FA composition of the recently consumed meal.

In conclusion, our data demonstrate that the FA composition of plasma TG and CE does not reflect short-term (i.e. previous weeks) dietary FA intakes, and that the FA composition of plasma CE may be the lipid fraction to utilise as an objective biomarker when investigating recent dietary FA intakes over this period. In addition, we show that the consumption of a high-fat meal influences the FA composition of plasma TG, PL and CE over the course of the postprandial period, with responses appearing to be specific to FAs in the different lipid fractions. Thus, the FA composition of plasma lipid fractions during the postprandial period (i.e. 1–6 h post meal) may not reflect fasting values. Based on these observations, it would be prudent to suggest that fasting blood samples should be utilised when using FA composition as a biomarker of dietary FA intake.
